# A summary of eye-related visits to a tertiary emergency department

**DOI:** 10.1038/s41598-021-83351-5

**Published:** 2021-02-15

**Authors:** Ravneet S. Rai, Nitish Mehta, Ryan Larochelle, Siddarth Rathi, Joel S. Schuman

**Affiliations:** Department of Ophthalmology, New York University School of Medicine, 550 1st Avenue, New York, NY 10016 USA

**Keywords:** Medical research, Health services

## Abstract

Timely ophthalmologic consultation is important to ensure patients receive high quality ophthalmologic care in the Emergency Department (ED). Tele-ophthalmology may prove useful in safely and efficiently managing ED eye-related complaints. Prior to implementing such a solution, current consultation patterns must be understood. We aimed to assess case-mix acuity and consultation workflow patterns in the ophthalmology consult service at a tertiary emergency department in New York City. The medical records of patients with eye-related complaints who presented to the ED between January 1, 2015 and December 31, 2015 were reviewed. Visits were retrospectively assigned acuities and the ophthalmologic subspecialty involved in the case was recorded. The number of ophthalmologic consultations ordered and consultant response times were analyzed. Patients who were transferred to the ED for eye-related complaints were included. The ED received 1090 eye-related complaints in this period. 60% were retrospectively assigned low acuity, 27% were assigned medium acuity, and 13% were assigned high acuity. Ophthalmology was consulted on 19% of low, 18% of medium, and 48% of high acuity cases. 44% of complaints involved the anterior segment and 30% involved oculoplastics. 2/3 of transfer patients initially assigned high acuity were downgraded to low acuity upon examination. On average, the consult note was created and signed within 109 and 153 min, respectively, after consult order. ED consults are heavily weighted towards pathology of low-to-medium acuity affecting the anterior segment and ocular adnexa. Currently available tele-ophthalmology technology can potentially address a large volume of eye-related visits.

## Introduction

Access to timely ophthalmologic consultation is important in the emergency department (ED). Some ophthalmologic complaints can be vision-threatening within a period of hours, while others can cause great discomfort to patients despite being non-emergent. Full ophthalmologic examination requires specialized knowledge and equipment, such as a slit lamp, fluorescein dye, fundus ophthalmoscopy, and ultrasound. Resource limitations dictate a need for appropriate utilization of in-person ophthalmologic consultations.

We herein perform a descriptive analysis of patients presenting with primary ophthalmologic complaints to a tertiary emergency department in New York City that has approximately 88,000 visits per year. We evaluate case mix acuity and consultation workflow patterns for these patients. The purpose of this analysis is to elucidate ophthalmologic urgent care needs and serve as the basis for designing telemedicine solutions to address the needs and pain points in delivering ophthalmologic care in the acute setting.

## Methods

Electronic medical records of all patients with eye-related complaints presenting to the ED from January 1, 2015 to December 31, 2015 were reviewed. All cases with an eye-related ICD-10 code as the primary diagnostic code were included in the analysis. Transfers from outside hospitals to the ED were included. Initial ED-assigned acuity by Emergency Severity Index (ESI) of 1 (most serious) to 5 (least serious) for each case was recorded. These cases were then retroactively assigned an ophthalmologic acuity (low, medium, or high) by the study team. Two individuals independently created an acuity scale based on common ophthalmology diagnoses based on currently accepted standards of practice, using American Academy of Ophthalmology Preferred Practice Guidelines as a generalized rubric. Any differences between the two individuals or equivocal diagnoses were adjudicated by a senior third member. A single individual reviewed each case and assigned the acuity level based on the diagnosis obtained by the consulting physician at the time of service.

This study was approved by the Institutional Review Board of the medical school associated with the ED in question. The IRB determined that informed consent was not necessary to access the medical records of the subjects of this study, as this was a large database study with full anonymization and no interventions performed. The collection and evaluation of protected patient health information was HIPAA compliant. This report adheres to the ethical principles outlined in the Declaration of Helsinki as amended in 2013.

Low acuity cases were defined as those that could be managed by ED staff with or without the assistance of an onsite ophthalmology resident physician or ophthalmologist. Such cases included corneal abrasions, viral conjunctivitis, or chalazion. Medium acuity cases were defined as those that required onsite ophthalmology evaluation and management, but did not require inpatient admission or acute surgery. Such cases included corneal foreign bodies, vitreous hemorrhage, and corneal ulcers. High acuity cases were defined as those that required emergent surgery or inpatient admission. Such cases included ruptured globe, macula-on retinal detachment, and orbital cellulitis. Each case was further divided into its most relevant ophthalmologic subspecialty: anterior segment, neuro-ophthalmology, oculoplastics, retina/posterior segment, and all others.

After the cases were categorized as described, the primary outcome variables were calculated. These variables were: percentage of cases in each retrospectively-assigned ophthalmologic acuity group (low, medium, or high) that resulted in ophthalmology consult; percentage of cases in each ED-assigned ESI acuity group (1–5) that resulted in ophthalmology consult; breakdown of cases by ophthalmologic subspecialty; and ophthalmology consult response times.

For statistical analysis, Chi-square tests were used to detect association between consultation rates and retrospectively assigned ophthalmologic acuity group, initial ED-assigned acuity group, and ophthalmologic specialty. A Kruskal–Wallis test was used to detect if there was a significant difference in time to note creation and note signature among retrospectively-assigned ophthalmologic acuity groups.

## Results

**Figure 1:**
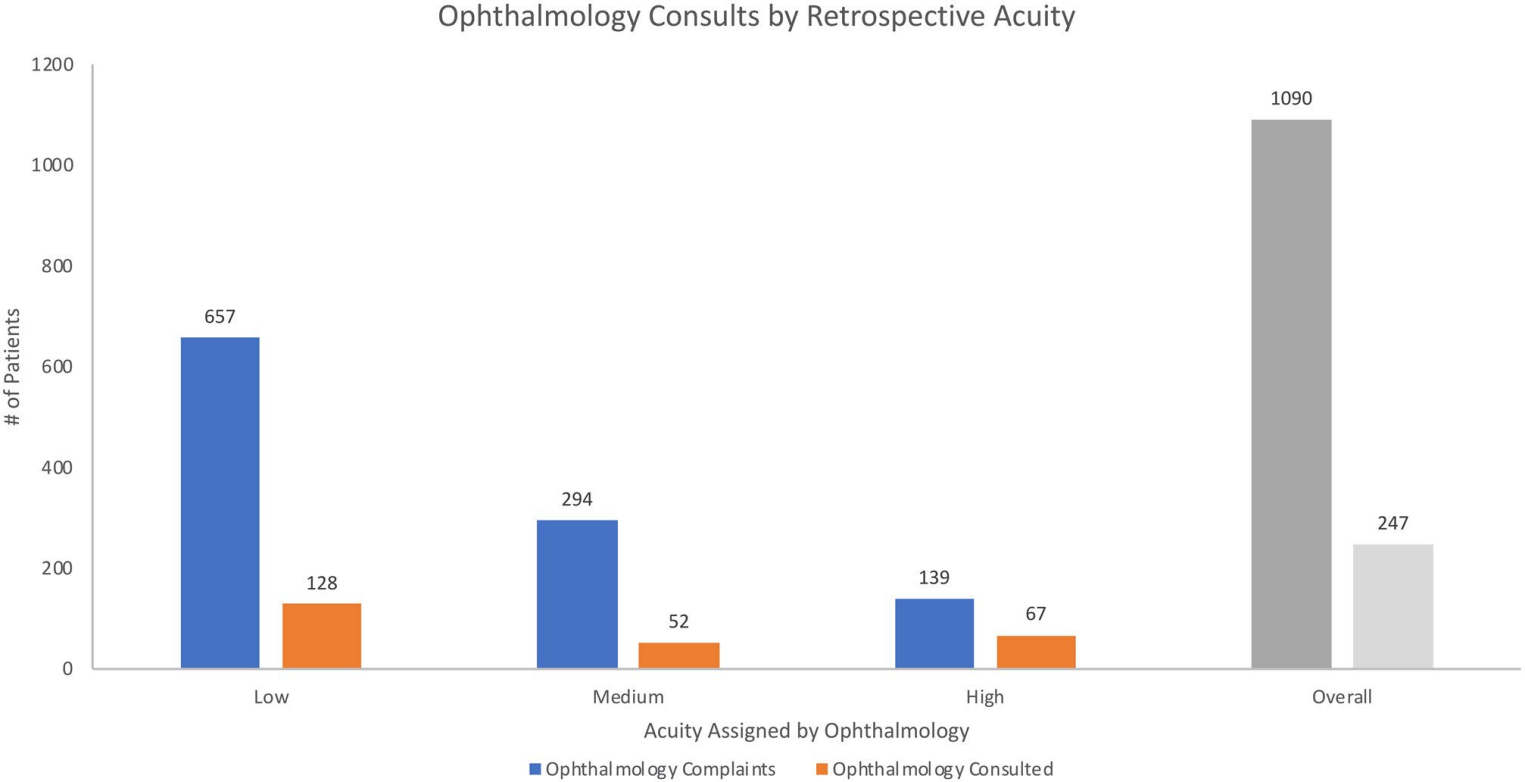
Breakdown of ophthalmology complaints and consults by retrospectively-assigned ophthalmologic acuity.

During the study period, the ED received 1090 primarily eye-related visits. The research team retrospectively assigned acuity levels to cases based on the ICD-10 diagnosis code for the ED encounter. Of these cases, 60% were retrospectively assigned as low acuity, 27% were assigned medium acuity, and 13% were assigned high acuity. Ophthalmology was consulted on 19% of the low acuity cases, 18% of the medium acuity cases, and 48% of the high acuity cases. In total, ophthalmology was consulted on 23% of all cases (Fig. 1).

Consultation rates were associated with retrospectively-assigned ophthalmologic acuity (p < 0.01, χ^2^).

**Figure 2:**
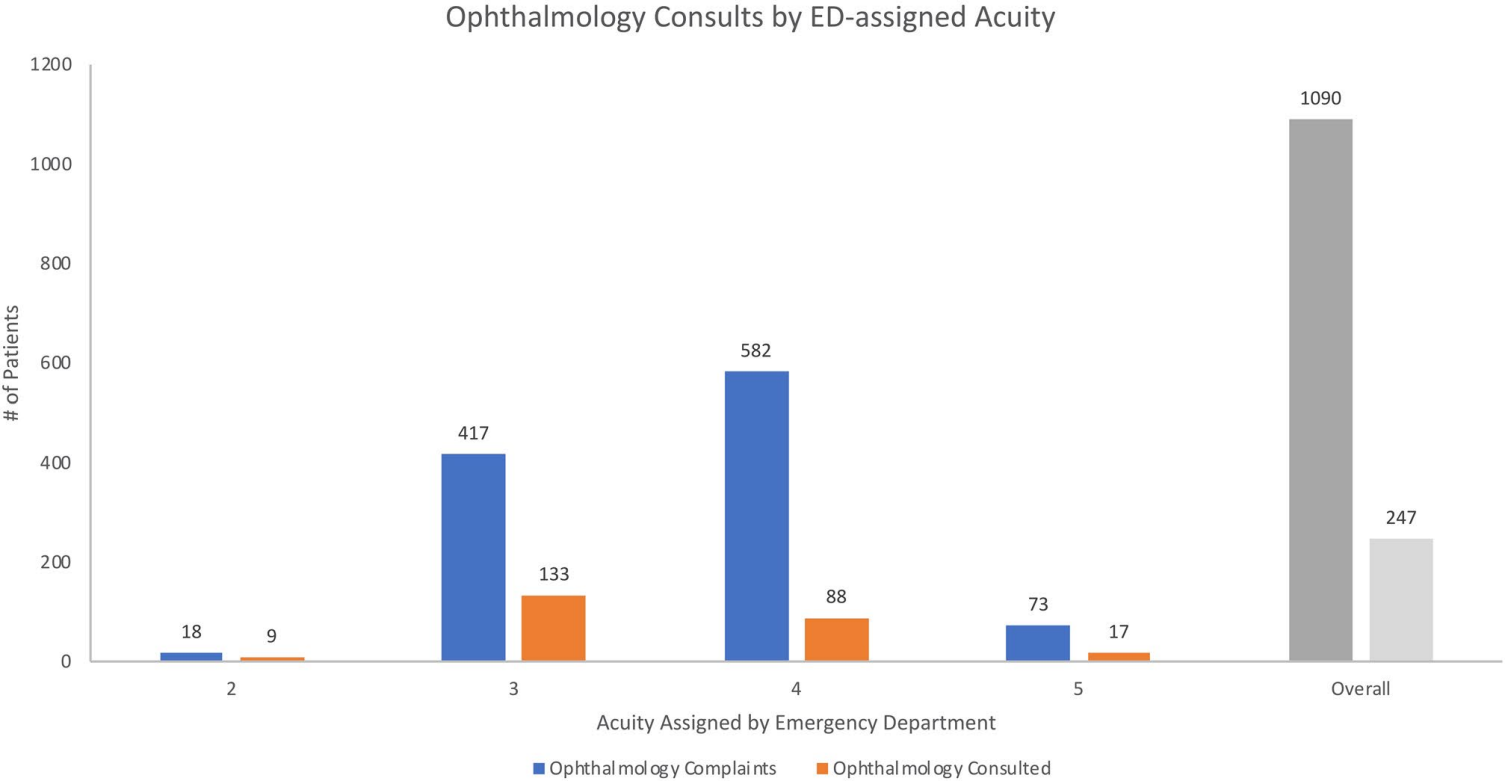
Breakdown of ophthalmology complaints and consults by initial ED-assigned acuity.

With regard to the initial Emergency Severity Index acuity (1–5) assigned by the ED prior to ophthalmologic consultation, no case was assigned an acuity of 1. The majority of cases fell within ESI acuities of 3 or 4, with notably fewer at the extremes of 2 or 5 (Fig. 2). Regardless of the ED-assigned ESI acuity, the majority of cases were retrospectively assigned low ophthalmologic acuity by the study team (ranging from 56% for ESI 2 and 3 to 67% for ESI 5). Even among ESI 2 cases, only 33% were retrospectively assigned high ophthalmologic acuity by the study team. It is important to note, however, that the ED assigns acuity based on the patient’s overall status, and not solely on the eye-related complaint.

Consultation rates were associated with initial ED-assigned acuity (p < 0.01, χ^2^).

During the study period, 9 patients were transferred to the ED from outside hospitals with ophthalmologic complaints. Three patients were assigned an ESI acuity of 1 by the ED, three were assigned an acuity of 2, and three were assigned an acuity of 3. Notably, of the three patients initially assigned an acuity of 1, two had non-emergent complaints after examination by the ophthalmology resident or attending and required outpatient follow-up (Table 1).

**Table 1:** Descriptions and outcomes of transfers to the ED from outside hospitals. The timing section refers to: minutes between triage and transfer note/consult note creation/consult note signature.

Complaint	Acuity	Time in minutes	Outcome	Procedure
Pain, foreign body	1	67/174/262	F/u primary ophthalmologist	No
Pain, photophobia	1	116/324/391	Conjunctivitis, f/u primary	No
Fx, red, blurry, pain	1	227/513/533	Admitted EtOH w/d; outpt. f/u	No
Laceration	2	No ophthalmology consult	F/u w/ plastics for suture removal	Sutures
Pain, blurry, hyphema	2	42 min until ophthalmology consult	Meds; f/u w/ primary	No
Lid laceration	2	Unavailable	Admitted for sedation and repair	No
Loss of vision s/p fall	3	108/633/677	Discharge; outpt. f/u	No
Preseptal abscess	3	106 min until ophthalmology consult	ED Obs for 48 h, f/u oculoplastics	No
Ophthalmoplegia, ataxia	3	No ophthalmology consult	Admitted to neuro; dx Miller–Fischer	No

**Figure 3:**
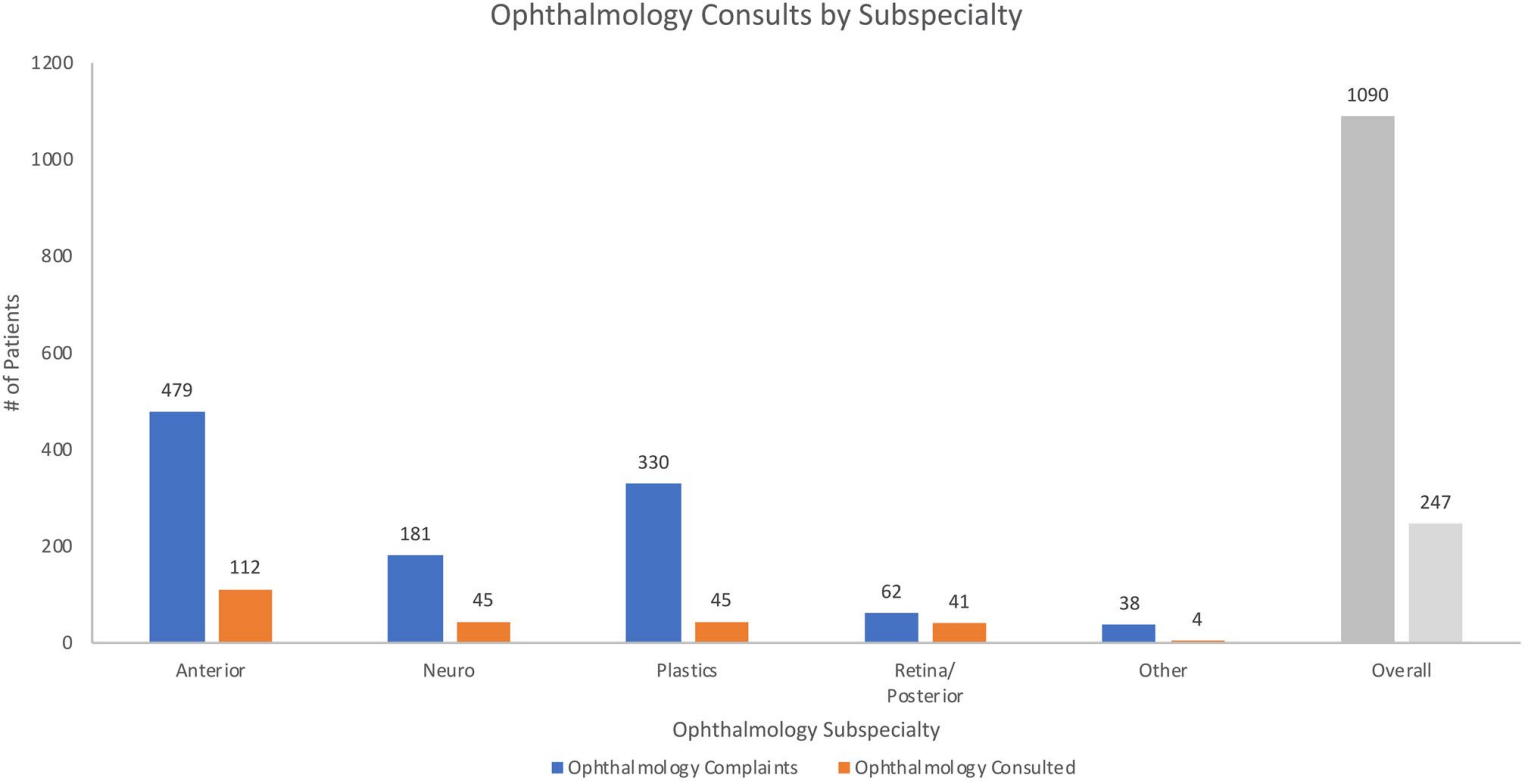
Ophthalmology consults by ophthalmologic subspecialty.

Significant differences in consultation patterns were noted based on the ophthalmologic subspecialty involved (Fig. 3). The largest proportion of diagnoses involved the anterior segment; however, cases involving the retina/posterior segment had the highest rate of ophthalmologic consultation. Overall, consultation rates were associated with ophthalmologic subspecialty (p < 0.01, χ^2^).

Among all cases, the median time to note creation was 109 min and the median time to note signature was 153 min. A Kruskal–Wallis test revealed that there was no difference among the three retrospectively-assigned ophthalmologic acuities in time to note creation (range 104–120 min, p = 0.70) and note signature (range 148–160 min, p = 0.65).

## Discussion

Ophthalmologic consultation rates at this tertiary ED are significantly associated with retrospectively-assigned ophthalmologic acuity, initial ED-assigned acuity, and ophthalmologic subspecialty. Consultation response times are not significantly associated with retrospectively-assigned ophthalmologic acuity. The majority of eye-related complaints that present at this ED are non-emergent. Consultation times can be lengthy, possibly due to provider resource limitations. Importantly, ED-assigned acuity and retrospective assigned acuity are often not in concordance.

Our study demonstrates similar results to other studies within the United States and elsewhere. A study by Channa, Zafar, Canner, Haring, Schneider, and Friedman (2016) using nationally representative data from the US Nationwide Emergency Department Sample found that 41.2% of ophthalmologic eye complaints in the ED are emergent and 44.3% are nonemergent^1^. Oftentimes, diagnoses for these non-emergent visits include viral conjunctivitis, dry eye syndrome, and corneal abrasion^1,2^. Our results from Manhattan, New York show a large volume of non-emergent complaints, although it is important to note there may be significant variations even within a relatively small area. For example, at a Level 1 trauma center in Brooklyn, New York, ophthalmologic consultations skewed heavily towards traumatic ocular complaints, including orbital wall fractures and traumatic iritis^3^. Other institutions utilizing our results to help refine their ophthalmology consultation programs must consider local variations.

Studies performed at a 24-h referral eye emergency service in Ireland and at an emergency department in the United Kingdom found that the most common diagnoses of referred patients were related to the anterior segment, similar to our results^4,5^. Notably, patients presenting with more serious ocular complaints typically presented after normal working hours^4^. Further studies will need to be performed in our study population to assess the effect of time of presentation to the ED on consultation rates and response times.

One important result of our analysis was the discordance between ED-assigned acuity and ophthalmology-assigned acuity in patients with an ocular complaint as the primary diagnosis. ED-assigned acuity status can be used to derive quality statistics or set hospital policy regarding consultations^6,7^. Our data demonstrate that retrospective ophthalmology-assigned acuity was somewhat discordant from ED-assigned acuity, particularly on the extremes of the ED Severity Index. For cases with ED-assigned acuity of 2, the second most acute level on the ED Severity Index, 55.6% were retrospectively assigned low ophthalmologic acuity by the study team. For cases with ED-assigned acuity of 5, the least acute level on the ED Severity Index, 13.6% were retrospectively assigned high ophthalmologic acuity by the study team. This discordance is likely due to the fact that ESI considers factors outside of the primary diagnosis, including vital signs, pain scores, and concomitant medical comorbidities. This suggests that ED assigned acuity should not be used to design and refine ophthalmology consultation programs in the future.

The overall dominance of low-acuity consultations and long wait time begets the question whether a tele-medicine solution may be able to improve the patient experience in emergency departments while maintaining quality. Telemedicine has been shown to improve resource utilization and decrease cost of treatment^8,9^. Patient satisfaction with ED treatment is often related to wait times^10^, and telemedicine has the ability to improve wait times^11^. Most importantly, tele-medicine has been shown to be safe^12^.

Telemedicine increases access to specialists for second opinions^13^. During critical events, such as multiple traumas or acute myocardial infarctions, the availability of telemedicine gives overstretched ED staff support to focus on the most urgent issues^13^. Telemedicine has also been shown to decrease time to care and improve care coordination^13^.

Telemedicine in ophthalmology, known as tele-ophthalmology, has been used successfully in a number of different contexts. It has been used in outpatient clinics to accurately diagnose and treat chronic blurry vision, screen for diabetic retinopathy, and evaluate age-related macular degeneration^14–16^. Tele-ophthalmology has also been used in the ED setting, though it is primarily implemented in rural EDs with lack of access to in-house ophthalmologists. In a rural Welsh hospital, a digital slit lamp camera connected via videoconferencing to an offsite specialist decreased the need for emergent patient transfers to other hospitals^17^. In remote areas of Australia, ED tele-ophthalmology programs have been in place for decades. For instance, in rural Queensland, a tele-ophthalmology platform using a slit lamp and high-resolution camera was in use as early as 1997. Implementation of this program resulted in no adverse outcomes and a significant decline in transfers to other hospitals^18^.

In Israel, a specialized ophthalmologic emergency room in a large hospital completed a feasibility study to test a tele-ophthalmology program that used a video camera attached to a slit lamp^19^. Emergency medicine residents would communicate with a senior ophthalmologist via telephone and relay the video of the slit lamp exam through a computer. The senior ophthalmologist would give the resident their diagnostic impression and management plan. Each case was then examined in-person by the attending ophthalmologist to determine the accuracy of the tele-ophthalmology diagnosis. Of the 49 patients (98 eyes) included in the study, the tele-ophthalmology diagnoses agreed with the in-person diagnoses 100% of cases. There was also a high level of patient satisfaction with the tele-ophthalmology program. These programs provide the blueprint for potential implementation in urban centers.

There are no ongoing tele-ophthalmology programs in metropolitan, high-resource, tertiary care EDs in the United States that have been described in the literature to our knowledge. Due to an overwhelming number of low-acuity complaints and lengthy consultation times, the ophthalmology consultation patterns at this ED suggest an opportunity to implement such a program to increase the efficiency of healthcare delivery.

## Conclusions

Ophthalmologic consultations at a tertiary ED in New York City vary by acuity and subspecialty. ED consults are weighted towards pathology affecting the anterior segment and ocular adnexa. Of patients transferred from outside facilities, cases marked initially as high acuity are often revised as low acuity after ophthalmologic consultation. Time to consultation note is not significantly related to case acuity, but is generally long. A telemedicine solution may improve the efficiency of triage, diagnosis, and management of these complaints.

## Limitations

We did not obtain information about the timing of presentation to the ED (e.g. after office hours, weekends) and did not assess how this may affect acuity and consultation patterns. We anticipate future studies addressing this in a prospective fashion.

## Data Availability

The datasets generated during and/or analyzed during the current study are available from the corresponding author on reasonable request.

## References

[1] Channa R, Zafar SN, Canner JK, Haring RS, Schneider EB, Friedman DS (2016). Epidemiology of eye-related emergency department visits. JAMA Ophthalmol..

[2] Sridhar J, Isom RF, Schiffman JC, Ajuria L, Huang LC, Gologorsky D (2018). Utilization of ophthalmology-specific emergency department services. Semin. Ophthalmol..

[3] Rizzuti AE, Vastardi M, Hajee M, Lazzaro DR (2013). Scope of resident ophthalmology consultation service and patient follow-up rates at a level 1 trauma center in Brooklyn, New York. Clin. Ophthalmol..

[4] Vartsakis G, Fahy G (2014). The profile of patients attending a triaged eye emergency service. Ir. J. Med. Sci..

[5] Bhopal RS, Parkin DW, Gillie RF, Han KH (1993). Pattern of ophthalmological accidents and emergencies presenting to hospitals. J. Epidemiol. Community Health.

[6] Hinson JS, Martinez DA, Cabral S, George K, Whalen M, Hansoti B (2018). Triage performance in emergency medicine: A systematic review. Ann. Emerg. Med..

[7] Villa S, Weber EJ, Polevoi S, Fee C, Maruoka A, Quon T (2018). Decreasing triage time: effects of implementing a step-wise ESI algorithm in an EHR. Int. J. Qual. Health Care.

[8] Zarca K, Charrier N, Mahé E, Guibal F, Carton B, Moreau F (2018). Tele-expertise for diagnosis of skin lesions is cost-effective in a prison setting: A retrospective cohort study of 450 patients. PLoS ONE.

[9] Wootton R (2001). Telemedicine. BMJ.

[10] Randhawa S, Viqar A, Strother J, Prabhu AV, Xia F, Heron D (2018). How do patients rate their radiation oncologists in the modern era: An analysis of Vitals.com. Cureus.

[11] Le LB, Rahal HK, Viramontes MR, Meneses KG, Dong TS, Saab S (2019). Patient satisfaction and healthcare utilization using telemedicine in liver transplant recipients. Dig. Dis. Sci..

[12] Taylor P (2005). Evaluating telemedicine systems and services. J. Telemedicine Telecare.

[13] Mueller KJ, Potter AJ, MacKinney AC, Ward MM (2014). Lessons from tele-emergency: Improving care quality and health outcomes by expanding support for rural care systems. Health Aff..

[14] Johnson Choon HT, Eugenie Wei TP, Sanjay S, Hock HT (2013). A pilot trial of tele-ophthalmology for diagnosis of chronic blurred vision. J. Telemedicine Telecare.

[15] Villa SR, Álvarez CA, Del Valle RD, Mendez RS, Garcia MC, Garcia MR (2016). Five-year experience of tele-ophthalmology for diabetic retinopathy screening in a rural population. Archivos de la Sociedad Española de Oftalmología (English Edition)..

[16] Kawaguchi A, Sharafeldin N, Sundaram A, Campbell S, Tennant M, Rudinsky C (2018). Tele-ophthalmology for age-related macular degeneration and diabetic retinopathy screening: A systematic review and meta-analysis. Telemedicine e-Health.

[17] Kulshrestha M, Lewis D, Williams C, Axford A (2010). A pilot trial of tele-ophthalmology services in north Wales. J. Telemedicine Telecare.

[18] Blackwell NA, Kelly GJ, Lenton LM (1997). Telemedicine ophthalmology consultation in remote Queensland. Med. J. Aust..

[19] Bar-Sela SM, Glovinsky Y (2007). A feasibility study of an Internet-based telemedicine system for consultation in an ophthalmic emergency room. J. Telemedicine Telecare.

